# Seismic velocity changes in the Groningen reservoir associated with distant drilling

**DOI:** 10.1038/s41598-022-22351-5

**Published:** 2022-10-20

**Authors:** Wen Zhou, Hanneke Paulssen

**Affiliations:** 1 Department of Earth Sciences, Utrecht University, Utrecht, The Netherlands; 2 Present Address: Department of Geoscience and Engineering, Delft University of Technology, Delft, The Netherlands

**Keywords:** Seismology, Geophysics

## Abstract

In this study, we show that passively recorded data of nearby passing trains by a deep borehole geophone array could be linked to fluctuations of the gas-water contact in the Groningen reservoir in The Netherlands. During a period of 1.5 months, changes of inter-geophone P-wave travel times were detected by deconvolution interferometry of the recorded train signals. P-to-S converted waves, obtained by deconvolution of the horizontal component by the vertical component at individual geophones, showed simultaneous variations. The observed travel-time changes could be related to fluctuations of the gas-water contact in the observation well caused by pressure variations at a well drilling 4.5 km away. The $\sim$ 3.5 day delay between drilling in the reservoir and the seismic response yields a hydraulic diffusivity of approximately 5 m$^2$/s and suggests that the pressure front is effectively propagated over such a long distance. Our observations illustrate that downhole geophone arrays can be used to monitor changes in the subsurface if repeating noise sources are available, and that unexpected effects may occur due to drilling.

## Introduction

A large number of studies have shown that changes in subsurface properties can be detected by seismic methods. Seismic velocity variations have been related to natural causes such as earthquake occurrence^1–3^, volcanism^4^, changes in groundwater level^5, 6^, or to human activity such as gas production^7^ or CO$_2$ injection^8^. In turn, the observed seismic velocity variations have been explained by changes in pore pressure^9, 10^, compaction^11, 12^ or gas-fluid substitution^13^.

It is well-known that the drilling of a deep well and fluid injection under high pressure can lead to induced seismicity^14^. A notable example is that of the Enhanced Geothermal System (EGS) in Pohang, Korea, which triggered a magnitude 5.5 earthquake^15, 16^. Fluid injection can also cause relatively distant seismicity (4–5 km), such as an earthquake swarm that was triggered by deep geothermal activities at Strasbourg, France^17^, or an earthquake swarm in the Groningen gasfield in The Netherlands that has been associated with mud losses during the drilling of a well^18, 19^. It is therefore relevant to monitor subsurface fluids.

**Figure 1:**
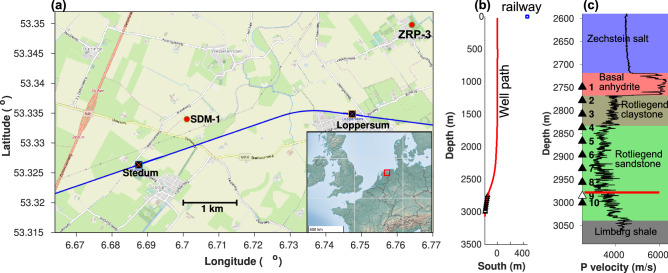
(**a**) Map of the area with the bottom locations of boreholes SDM-1 and ZRP-3, and the railway line with the villages Stedum and Loppersum. Inset shows the location of the Groningen gas field in Europe. (**b**) The SDM-1 well path with geophone positions. (**c**) Depths of the geophones and lithology. The P-wave speed from well-log data is shown in black and the horizontal red line indicates the level of the gas-water contact in the reservoir. Geophone 9 was out of order. (Figure a was created using MATLAB v2018 (https://www.mathworks.com) with data from the geobasemap and Open Street Map (https://www.openstreetmap.org)).

In this study, we present observations of seismic wave travel-time changes in the vicinity of a deep borehole geophone array in the Groningen gasfield^20^ that are related to drilling into the 3-km deep reservoir at 4.5 km distance. Temporal changes were detected with nearby passing trains, acting as repetitive noise sources. Figure 1 shows the setting, where SDM-1 is the well with an array of 10 geophones at 2.7–3 km depth and ZRP-3 is the well that was drilled by Nederlandse Aardolie Maatschappij (NAM) from May to the end of August 2015^21^. Figure 1a also shows the railway line between the villages Stedum and Loppersum at 0.5 km distance from observation well SDM-1, with trains passing twice per hour during the day in both directions.

We analysed the continuous geophone data for two 5-month deployments in 2015 (Jan–Jun and Jul–Dec) and extracted the train signals as described in a previous study^12^. Interferometry by deconvolution was applied to the train signals in order to detect temporal changes of the inter-geophone P-wave travel times, where a previous study focused on the minor effects of compaction. In addition, we also investigated temporal variations of P-to-S converted waves and changes in ambient noise level.

## Observations

### Travel-time changes of direct P waves

We applied interferometry by deconvolution^22, 23^ to train signals recorded on the vertical component to retrieve the P-wave responses between all geophone pairs in well SDM-1. The high-frequency content of the train signals (30–90 Hz) allowed accurate measurements of inter-geophone P-wave travel times, and the large number of passing trains ($\sim$ 60 per day) provided a large number of measurements. Details of the processing are described in a previous study^12^.

**Figure 2:**
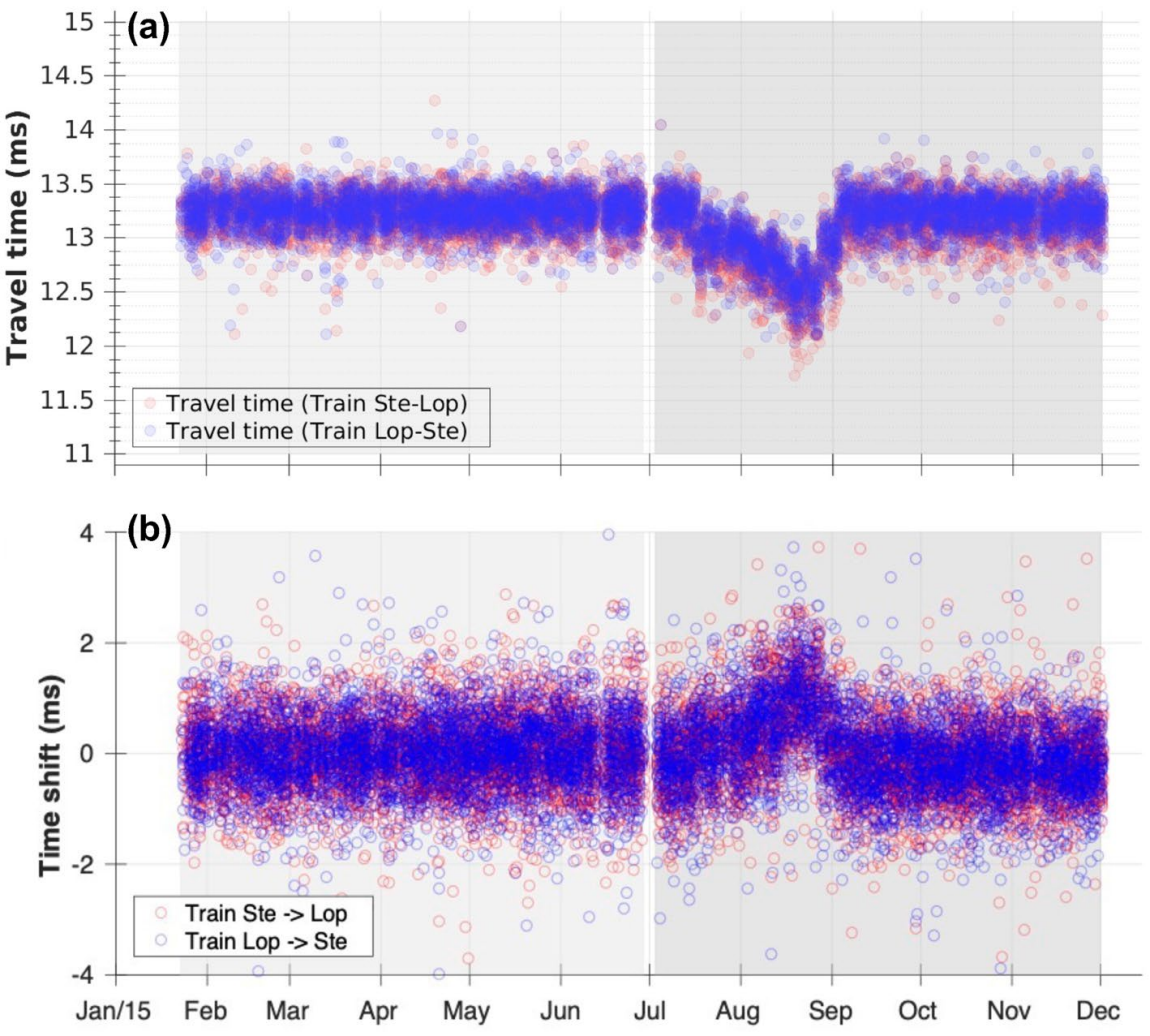
(**a**) P-wave travel times between geophone 8 and 10 measured by train signal deconvolution; red circles for trains from Stedum to Loppersum, blue circles for trains from Loppersum to Stedum. The graph for the first deployment period (Jan 23–Jun 29 2015) has a light-grey background, for the second deployment period (Jul 3–Dec 1 2015) the background is darker grey. (**b**) Time shifts of PS waves obtained from HZdecon responses at geophone 10.

The train signal deconvolutions showed a clear P-wave travel-time reduction from mid July to the beginning of September 2015 for all geophone pairs that included the lowermost geophone (geophone 10), whereas the P-wave travel times between other geophone pairs did not show such an anomaly (Supplementary Material, Section 1). Figure 2a presents the P-wave travel-time measurements between geophone 8 and geophone 10; the travel times ($t_P$) show a large travel-time deviation ($\Delta t_P$) of up to $\sim$ 0.7 ms from mid July to the beginning of September 2015.

To check if a geophone shift could cause the apparent P-wave travel-time changes, we investigated the ample high-frequency (up to 1000 Hz) acoustic signals that travelled within the borehole fluid. Deconvolution interferometry was applied to these signals (filtered at 100–300 Hz), which did not show associated travel-time changes in the corresponding period (Supplementary Material, Section 2).

Because the data from the train signals are sensitive to the seismic velocities around the borehole^12^, the observations are likely related to changes in the medium in the vicinity of geophone 10. In particular, we realized that geophone 10 is the only geophone beneath the gas-water contact (GWC) and the inter-geophone travel times to geophone 10 are sensitive to the level of the GWC. An upward movement of the GWC would replace gas by water in the porous sandstone, increasing the effective incompressibility and therefore the P-wave speed, leading to decreasing P-wave travel times. Bringing the GWC back to its original level, would bring the travel times back to their normal values again.

**Figure 3:**
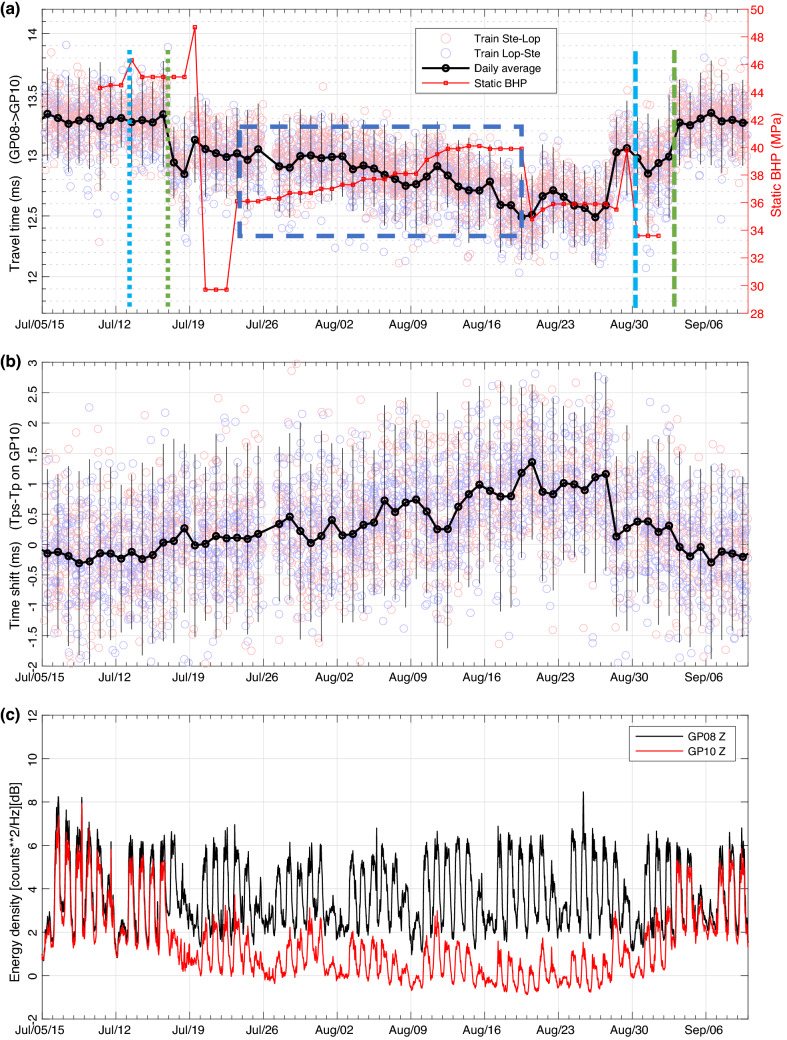
(**a**) P-wave travel times from geophone 8 to geophone 10 from single train deconvolutions (red and blue circles) and their daily average (black). The error bar is twice the standard deviation. Red squares indicate the static bottom-hole pressure ($BHP_s$) at ZRP-3 (courtesy NAM). $BHP_s$ represents only part of the bottom hole pressure (BHP) because dynamic effects are not included. Dotted and dashed vertical blue lines indicate the start time of drilling into the Ten Boer claystone and the end time of the well operations, respectively. Dotted and dashed vertical green lines indicate the start and end times of the seismic anomaly as inferred from the noise recordings (Fig. 4). The dashed blue box outlines the anticorrelation between the bottom hole pressure and P-wave travel time. (**b**) Travel-time shifts of PS waves at geophone 10 measured from the HZdecon responses with error bars of two times the standard deviation. (**c**) Average power density (5–30 Hz) in dB for geophone 8 (black) and 10 (red) smoothed over a 1-h time window.

The data of Fig. 2a are shown again in Fig. 3a for the period from July 5 to September 10, 2015. The P-wave travel times for the single train deconvolutions are indicated by the blue and red circles and their daily average by the black circles with an error bar of two standard deviations. It is clear that the travel-time anomaly starts on July 17 and ends on September 3. It was found that this time span approximately matched the drilling of the reservoir of well ZRP-3 at 4.5 km distance and that there might be a relation between the two.

### Travel-time changes of P-to-S converted waves

Similar to the P-wave travel times, we attempted to measure S-wave travel-time changes by train signal deconvolution. This was not feasible because the S-wave responses, obtained by deconvolution of the horizontal components between geophone pairs, had a poor signal-to-noise ratio. We therefore used an alternative approach which is similar to the classical receiver function approach^24^, albeit in a reversed setting because the receivers are at depth and the incoming wave field is produced by trains at the surface. The deconvolution of the horizontal component by the vertical component (HZdecon) for a single geophone gives the response of P-to-S (PS) converted waves generated at lithological contrasts within the overburden. A PS wave converted at an interface in the overburden produces a peak in the HZdecon response at the time difference between the (converted) S wave and the direct P wave, in other words, at the S-P travel-time difference from the interface to the receiver ($t_{PS}-t_P$). The method and interpretation is described in the Supplementary Material (Section 3), here we present the time-lapse observations.

No significant temporal changes of the HZdecon responses were detected for the upper geophones, but the HZdecon responses of geophone 10 showed a time shift from mid July to the beginning of September. To quantify the observed time shift, we cross-correlated the HZdecon responses for each of the train signals with the average of the entire 5-month deployment period. The wavelet of the PS wave generated at the anhydrite layer just above the claystone-sandstone reservoir was selected for the cross-correlation. The time shifts of this wavelet for the individual train signals are presented in Figs. 2b and 3b shows their daily average with a two-standard deviation error bar.

It is obvious that the time shifts of the PS waves at geophone 10 occur in exactly the same span as the travel-time anomalies of the direct P waves to geophone 10 and that the two types of data appear to be anticorrelated. The observed $\sim$ 0.7 ms decrease in P-wave travel time ($\Delta t_P$) together with the to $\sim$ 1 ms increase in the delay time of the PS waves ($\Delta (t_{PS}-t_P)$) suggest a P-wave travel-time decrease of $\sim$ 0.7 ms accompanied by a small S wave travel-time increase ($\Delta t_S \simeq 0.3$ ms). Note that these numbers are indicative, because their uncertainties are large, particularly for the PS waves (Fig. 3a,b). The observations support the hypothesis of a rise of the gas-water contact, because the substitution of gas by water in the porous sandstone would increase the P velocity and slightly decrease the S velocity^25, 26^.

### Changes in ambient noise level

**Figure 4:**
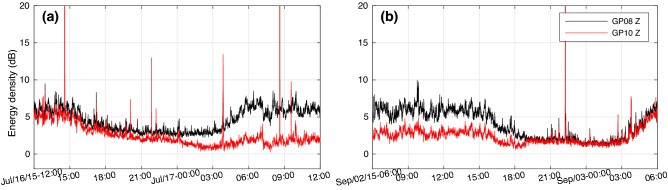
Vertical component noise level of geophone 8 (black) and 10 (red) for the 5–30 Hz frequency band (**a**) at the beginning, and (**b**) the end of the anomalous period.

The changes in the train-signal responses of geophone 10 prompted a more thorough analysis of the noise recorded by this geophone. We calculated the average power density in the 5–30 Hz frequency band for consecutive 30 s time windows of the continuous vertical component noise data of geophone 8 and 10. Note that this frequency band is lower than that of the train signals used in the P-wave interferometry and PS wave studies. Figure 3c shows the results after applying a 1 h moving average to reduce the peaks in some of the 30 s time windows due to earthquakes. Daily and weekly patterns are evident with a discrepancy in the noise levels between the two geophones for the time span between July 17 and September 2. During this period the noise level of geophone 10 is clearly lower. Figure 4 shows that the noise level data allowed accurate timing of the beginning (July 17, 00 h) and the end (September 2, 19 h) of the anomalous time span. Interestingly, a nearby micro-earthquake was detected at the beginning of the period suggestive of a local stress change (Supplementary Material, Section 4).

## Interpretation

As mentioned before, the temporary decrease in P-wave travel time to geophone 10 and the increase in the delay time of the PS waves might be explained by a temporary upward movement of the gas-water contact (GWC). Here we investigate this interpretation in more detail.

### Elevation of the GWC from seismic observations

The change in seismic velocities due to the substitution of gas by water in a porous sandstone can be calculated with the Gassmann model of fluid subsititution^25, 26^. The bulk modulus of a fluid saturated rock is related to the porosity and to the bulk moduli of the mineral matrix, the pore fluid and the dry rock frame. The bulk modulus of the fluid will increase in case of gas-water substitution, and this will increase the effective bulk modulus of the rock, and therefore the P velocity. The shear modulus, on the other hand, will not change as it is mainly dependent on the solid frame of the rock. However, due to the small increase in density, a slight decrease in S velocity is expected.

A quantitative estimate of the change in the level of the GWC using Gassmann’s model would require accurate values of the bulk and shear moduli of the matrix, fluids (gas and brine) and rock frame for the local rock. Because these are unknown and rough estimates would have large uncertainties, we took a more practical approach. We estimated the average P velocity above and below the GWC from the sonic log data and found P velocities of 3321 m/s and 3688 m/s, respectively (Supplementary Material, Section 5). Assuming these values, a 0.7 ms P-wave travel-time decrease would correspond to an elevation of the GWC by 23 m. We further checked if this elevation of the GWC could also explain the increase in the PS delay time ($\Delta (t_{PS}-t_P)$ $\simeq$ 1.0 ms). Assuming a 23 m shift of the GWC, an S-wave travel-time increase of 0.3 ms, vertical propagation with an S wave velocity of 2000 m/s for the sandstone with gas^12^, we find an S wave velocity decrease of only 52 m/s (2.6%). This decrease would be solely the effect of the density increase on the shear velocity caused by the replacement of gas by water. Although the values appear realistic, it should be noted that the uncertainties are large and that 23 m should only be interpreted as an indication of GWC elevation inferred from our measurements.

The other observation is the rapid decrease in the noise level, as well as its rapid return to the normal level observed for geophone 10 relative to other geophones (Fig. 4). Such rapid changes are easily accomplished by changes in the level of the GWC, although it is not clear how this would reduce the noise level.

### Relation with borehole operations at borehole ZRP-3

If a temporary elevation of the GWC can explain the seismic observations, the question arises what caused the elevation of the GWC. The gas production data in the area were checked, but do not show a correlation with our data. Since the timing of the anomaly appeared to be correlated to the drilling of well ZRP-3 at 4.5 km distance, we investigated the detailed drilling report that was provided by NAM.

Drilling started on May 23 (2015) and the reservoir was reached by drilling into the Ten Boer claystone on July 13 (Fig. 3a). Downhole losses of the drilling mud occurred on July 18 and the first hours of July 19. Deeper drilling took place during limited periods on single days between July 23 and August 21 when the maximum depth of 3284 m was reached. The depth of the GWC was reached on July 31, and the Carboniferous shale was drilled on August 11. Cementing of the borehole took place on August 28–29, and the borehole was left on August 30 after cement hardening.

The first conclusion is that there is no correlation between our observations and the intervals of actual drilling. Firstly, drilling and coring periods were scattered in time, while our observations show a month-long trend of mostly decreasing travel times (Fig. 3a). Secondly, drilling noise would affect the travel times between all the geophone pairs, but this is not observed (Supplementary material, Fig. S3). Thus, drilling noise cannot explain the observations. We also considered the downhole losses that occurred during drilling in the Ten Boer claystone. However, these downhole losses initiated 30 h after the start of the anomalous observations.

A more likely cause is the pore pressure variations caused by drilling. NAM provided us with data of the static bottom hole pressure ($BHP_s$), calculated from the borehole depth (*h*), the drilling mud density ($\rho _m$) and the gravitational acceleration (*g*): $BHP_s=\rho _m g h$. These data are shown in Fig. 3a. Note that $BHP_s$ represents only part of the total bottom hole pressure at the well head (BHP) because dynamic pressure effects are not included. Rapid decreases in $BHP_s$, for instance between July 19 and 23 related to mud losses, will have been dynamically compensated by borehole fluid circulation to stabilize BHP. From July 23 to August 19, when drilling depths increased from 2919 to 3267 m there is a gradual increase in $BHP_s$ from 36 to 39 MPa, as indicated by the dashed blue box in Fig. 3a. The gradual trend of increasing BHP is anti-correlated with the P-wave travel-time decrease from geophone 8 to 10 and correlated with the increase in PS delay time at geophone 10 (Fig. 3a,b).

Assuming that our anomalous observations at SDM-1 are related to the bottom hole pressure (BHP) at ZRP-3, it is likely that they are linked through changes in pore pressure. An elevation of the GWC of $\sim$ 23 m would correspond to an increase of the pore pressure in the water bearing part of the sandstone of $\sim$ 0.23 MPa ($\Delta P = \rho _w g \Delta h$). By linking the beginning and the end times of the drilling operations in the reservoir to the beginning and the end times of our anomalous observations, we calculated the time that the pressure front took to propagate from ZRP-3 to SDM-1. Drilling into the Ten Boer claystone took place on July 13, between 7:45 and 17:00, while the anomalous seismic observations started on July 17 at $\sim$ 00:00 (Fig. 4a). This gives a time delay of 3 days and 7–16 h. A similar calculation can be made for the end of the anomalous period. The hardening of the cement at ZRP-3 took place on August 30 (00:00–07:30). After cement hardening the well was sealed and there was no influence from the borehole operations anymore. Combining this to the end of the anomaly at SDM-1 on September 2 at 19:00 (Fig. 4b) gives a time delay of 3 days and 11.5–19 h.

### Pore pressure diffusion

From our seismic observations and their correlation with the bottom hole pressure at ZRP-3, it is inferred that pore pressure variations may have caused changes in the level of the GWC in SDM-1. Next, it should be verified that the process of pore pressure diffusion can explain the time delay between reservoir drilling at ZRP-3 and the GWC response at SDM-1 at 4.5 km distance.

In case of isotropic and spherical diffusion, the hydraulic diffusivity (*D*) associated with pore pressure diffusion in a fluid bearing porous medium can be estimated from the time (*t*) it takes the pressure front to reach a certain distance (*r*)^27^

1 $$\begin{aligned} r = \sqrt{4 \pi D t}. \end{aligned}$$

The pore pressure diffusivity is estimated from the propagation time of the pressure front, given the time delays of 3 days and 7–16 h (beginning) and 3 days and 11.5–19 h (end). The largest (3 days and 19 h) and smallest (3 days and 7 h) times give diffusivities of 4.9 m$^2$/s and 5.7 m$^2$/s, respectively.

An independent estimate of the hydraulic diffusivity (*D*) can be calculated from the material properties, including the average porosity (0.15^28^) and permeability (120 mD^29^) with further details provided in “Method”. We find a pore pressure diffusivity of 3.9 m$^2$/s, which is similar to, although somewhat smaller than, the previously estimated diffusivity range of 4.9–5.7 m$^2$/s. For this diffusivity range, permeabilities of 151–176 mD are required, somewhat higher than our adopted value of 120 mD, but within the wide range of 1–1000 mD measured for the Groningen gas reservoir^30^. Thus, it is concluded that pore pressure diffusion in the water-bearing part of the reservoir can explain the time delay between the overpressure at ZRP-3 caused by drilling and the change in the GWC at SDM-1.

**Figure 5:**
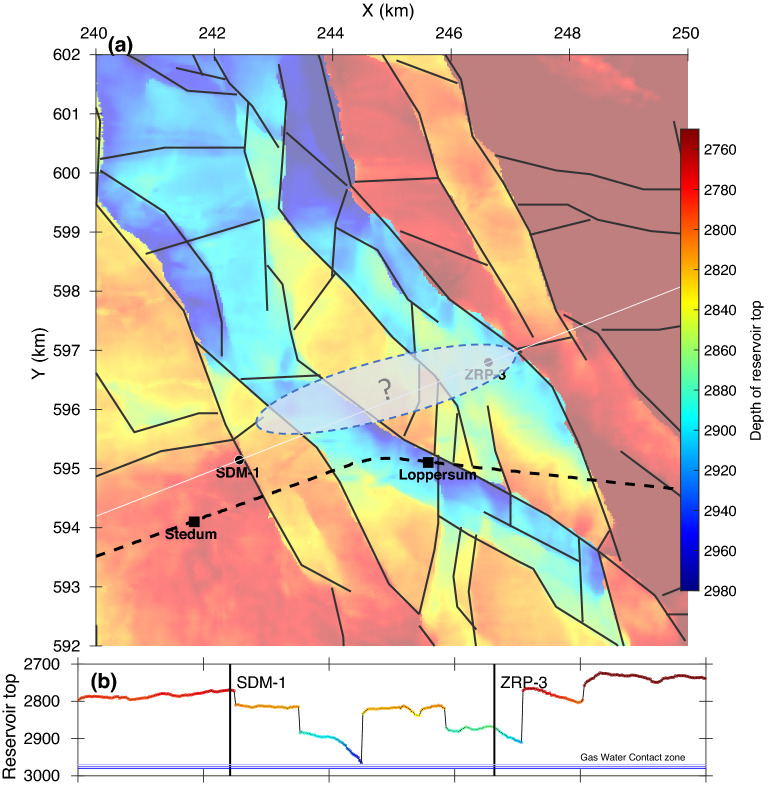
(**a**) Topography of the top of the reservoir with faults in black and the locations of boreholes SDM-1 and ZRP-3. The railway from Stedum to Loppersum is indicated by the dashed black line. The transparent region indicates a speculative connection between two fault segments on either side. (**b**) Depth of the top of the reservoir for the cross-section through SDM-1 and ZRP-3 (white line in a).

The Groningen reservoir is highly faulted, and faults may either act as barriers or as efficient conduits of pore pressure depending on the direction: the permeability is generally high within the damage zone parallel to the fault and low across the fault^31^. The NAM fault map for the top of the reservoir (Fig. 5a) shows a fault with an offset of approximately 150 m halfway between SDM-1 and ZRP-3 separating two compartments of the reservoir with the wells on either side (Fig. 5b) . This fault is likely to hinder direct pore pressure diffusion through the gas-bearing parts between the two compartments. On the other hand, the $\sim$ 20 m change in the level of the GWC at 4.5 km distance from the drilling location and the high diffusivity ($\sim$ 5 m$^2$/s) suggest a high permeability conduit between the two locations. The NAM fault map does not show a connecting fault, although, speculatively, there are two ENE-WSW trending fault segments that might be linked at the bottom of the reservoir (Fig. 5a).

It is important to know that SDM-1 is a well that is open at the top and bottom and perforated at reservoir depths between 2965 and 2995 m. The load of a high density brine column within the well prevents the gas of the reservoir from flowing inwards through the perforations. With the well being an open system, it is sensitive to variations in hydrostatic pressure in the reservoir. Our speculative hypothesis is that the pressure front, caused by overpressure at the remote well head and propagated through the water-bearing part of the reservoir, reached SDM-1 and moved the brine column up, elevating the water table within the well. Following the level change within the perforated well, the GWC in its immediate vicinity was also elevated which was detected by the seismic data.

Whereas we realize that parts of our interpretation are highly speculative, we were unable to find another plausible explanation. Nevertheless, it seems obvious that the observations are linked to distant drilling, an effect that is unexpected and may be important for other drilling activities.

## Conclusion

In this study, we have shown that temporal variations in seismic velocity at depth can be detected with a deep borehole geophone array and repetitive noise sources such as passing trains. Inter-geophone P-wave travel times along a borehole array in the Groningen gas reservoir, obtained by deconvolution interferometry of train signals, showed significant temporal variations over a period of 1.5 months. Responses of P-to-S converted waves, obtained by horizontal-vertical deconvolution showed similar, but anti-correlated temporal variations. Both types of observations can be explained by fluctuations in the level of the gas-water contact in the reservoir, and are related to drilling in the reservoir at 4.5 km distance. Pore pressure diffusion with a diffusivity of roughly 5 m$^2$/s is inferred from the time delay between the drilling activities and their seismic response. We speculate that the overpressure at the well head may have propagated along a permeable conduit such as a fault and that it may have affected the gas-water contact in the vicinity of the observation well.

Our study demonstrates that recordings of deep borehole geophone arrays, deployed for monitoring seismicity at depth as in this case, can be used to detect changes in material properties if repeating sources are available. In this study we used train signals as passive noise sources, but active sources can be used as well. The notion that (unexpected) changes of a gas-fluid interface may be inferred from geophone array data is also relevant for other types of subsurface monitoring, such as geothermal or CO$_2$ storage.

## Method

We calculated the hydraulic diffusivity (*D*) from the material properties^32^:

2 $$\begin{aligned} {D} = \frac{N k}{\eta } \end{aligned}$$

where *k* is the permeability, $\eta$ the pore fluid dynamic viscosity, and *N* a poroelastic modulus:

$$\begin{aligned} N= & {} MP_d/H \\ M= & {} (\phi /K_f + (\alpha - \phi )/K_g)^{-1} \\ H= & {} P_d+\alpha ^2M \\ \alpha= & {} 1 - K_d/K_g \\ P_d= & {} K_d + (4/3)\mu _d \end{aligned}$$

where $K_{f,d,g}$ is the bulk modulus of the fluid (*f*), dry rock frame (*d*) and the solid grain material (*g*), $\mu _d$ the shear modulus of the frame, and $\phi$ the porosity^27, 32^. The following values were adopted from the literature: *k* = 120 mD^29^, $\phi$ = 0.15 for the Groningen sandstone reservoir^28^; $K_g$ = 37 GPa for the bulk modulus of quartz^26^; $K_f$ = 2 GPa for the bulk modulus of water; $\eta$ = 2.8 10$^{-4}$ Pa s for the pore fluid dynamic viscosity of water at 100 $^{\circ }$C. The bulk and shear modulus of the dry rock frame were derived from the seismic velocities of the gas saturated sandstone ($V_P$ = 3300 m/s, $V_S$ = 2100 m/s)^12^ combined with the density from well log data ($\rho$ = 2200 kg/m$^3$), yielding $K_d$ = 11 GPa and $\mu _d$ = 9.7 GPa. With these values a diffusivity of 3.9 m$^2$/s is obtained.

## Supplementary Information

Supplementary Information.

## Data Availability

Seismic data used in this study are available from EPOS-NL, https://epos-nl.nl/.
