# Supplementary material for: Seismic velocity changes in the Groningen reservoir associated with distant drilling
Source: Sci Rep. 2022 Oct 20;12:17523. doi: 10.1038/s41598-022-22351-5 (PMC9585063; doi:10.1038/s41598-022-22351-5)
Supplement: Supplementary file 1 — Supplementary Information. [file 41598_2022_22351_MOESM1_ESM.pdf]

Supporting Information for:
Seismic velocity changes in the Groningen reservoir associated with distant drilling

Wen Zhou, Hanneke Paulssen

Contents

1 Time-lapse P-wave travel times by deconvolution interferometry of train signals 2

2 Time-lapse borehole fluid wave travel times by deconvolution interferometry 5

3 Time-lapse P-to-S converted wave travel times by Horizontal-Vertical deconvolution of train signals 9

3.1 Horizontal-Vertical deconvolution: HZdecon 9

3.2 Synthetic HZdecons 10

3.3 Observed train-signal HZdecons 11

3.4 Time-lapse train-signal HZdecons 11

4 Noise level and micro-earthquake 13

5 P-wave velocity above and below GWC 14

References 15

1 Time-lapse P-wave travel times by deconvolution interferometry of train signals

In this section we illustrate some of the time-lapse variations of the P-wave responses obtained by train signal deconvolution.

The 20-s duration train signals were detected from the continuous data using spectrograms as described in (13). Fig. S1 shows the waveforms of the direct P-wave (obtained by deconvolution of the train signal recorded by the top geophone) stacked per day (approximately 60 trains per day) for August 28, 2015, the day with smallest inter-geophone travel times to geophone 10 (red), and for November 3, 2015, an arbitrary day in the same deployment period. Note the similarity of the peak in the cross-correlations for the first 8 geophones and the subtle shift for the lowermost geophone (GP10).

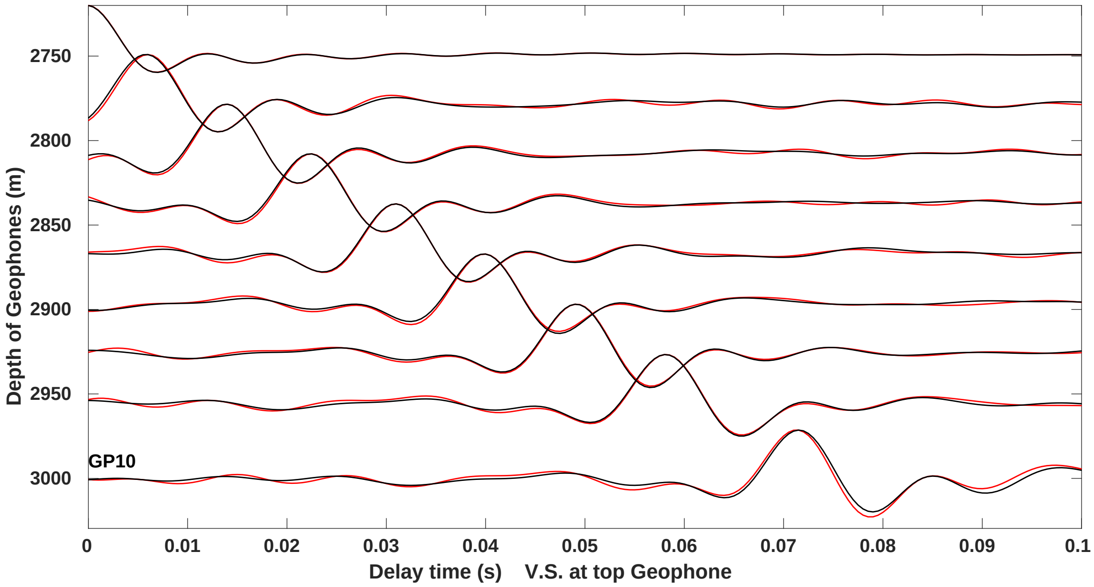

Figure S1: Daily stacked P-wave response by train-signal deconvolution interferometry: August 25, 2015 (red) and November 3, 2015 (black).

Fig. S2 shows the time-lapse variations of the daily stacked interferograms from geophone 8 to geophone 10 over the two deployment periods in 2015. The period with the anomalous observations (mid-July until September 1) is clearly visible.

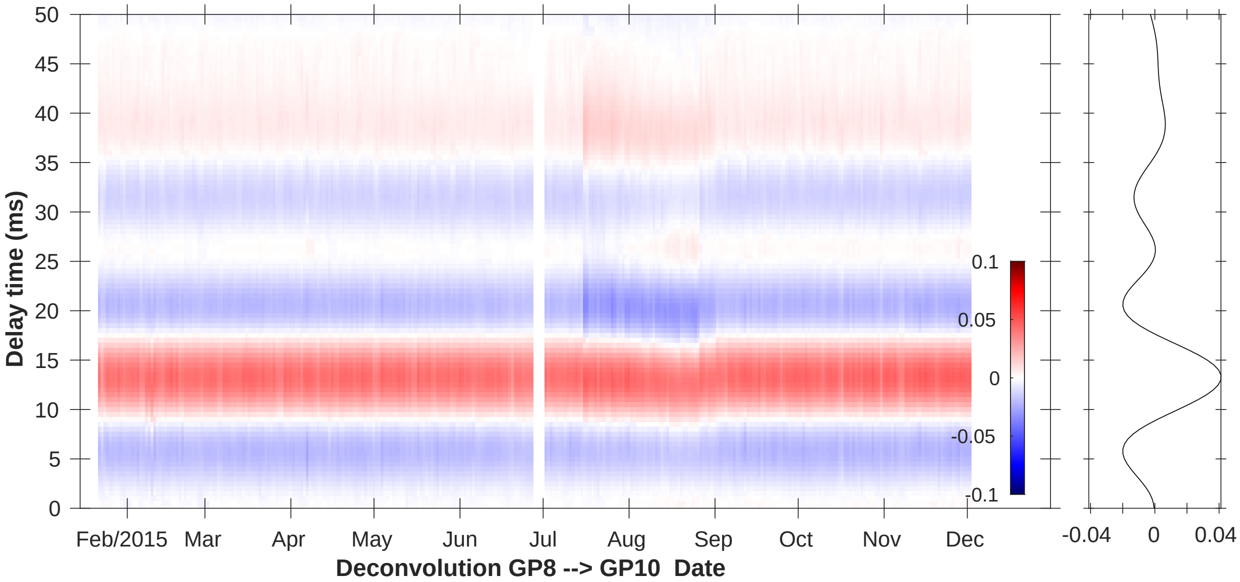

Figure S2: Left panel: Daily stacked deconvolution interferograms between geophone 8 (virtual source) and geophone 10 (virtual receiver) for trains from Stedum to Loppersum with colour-coded amplitudes. Right panel: Waveform stack over the first half-year deployment (Jan 23 - Jun 29, 2015).

Fig. S3 (next page) shows the travel-time measurements (at maximum of cross-correlation) from the daily stacked deconvolution interferograms for all geophone pairs. The measurements in blue are for trains from Stedum to Loppersum, the measurements in red are for trains in the opposite direction. The stacks are for ~30 trains per day in each direction.

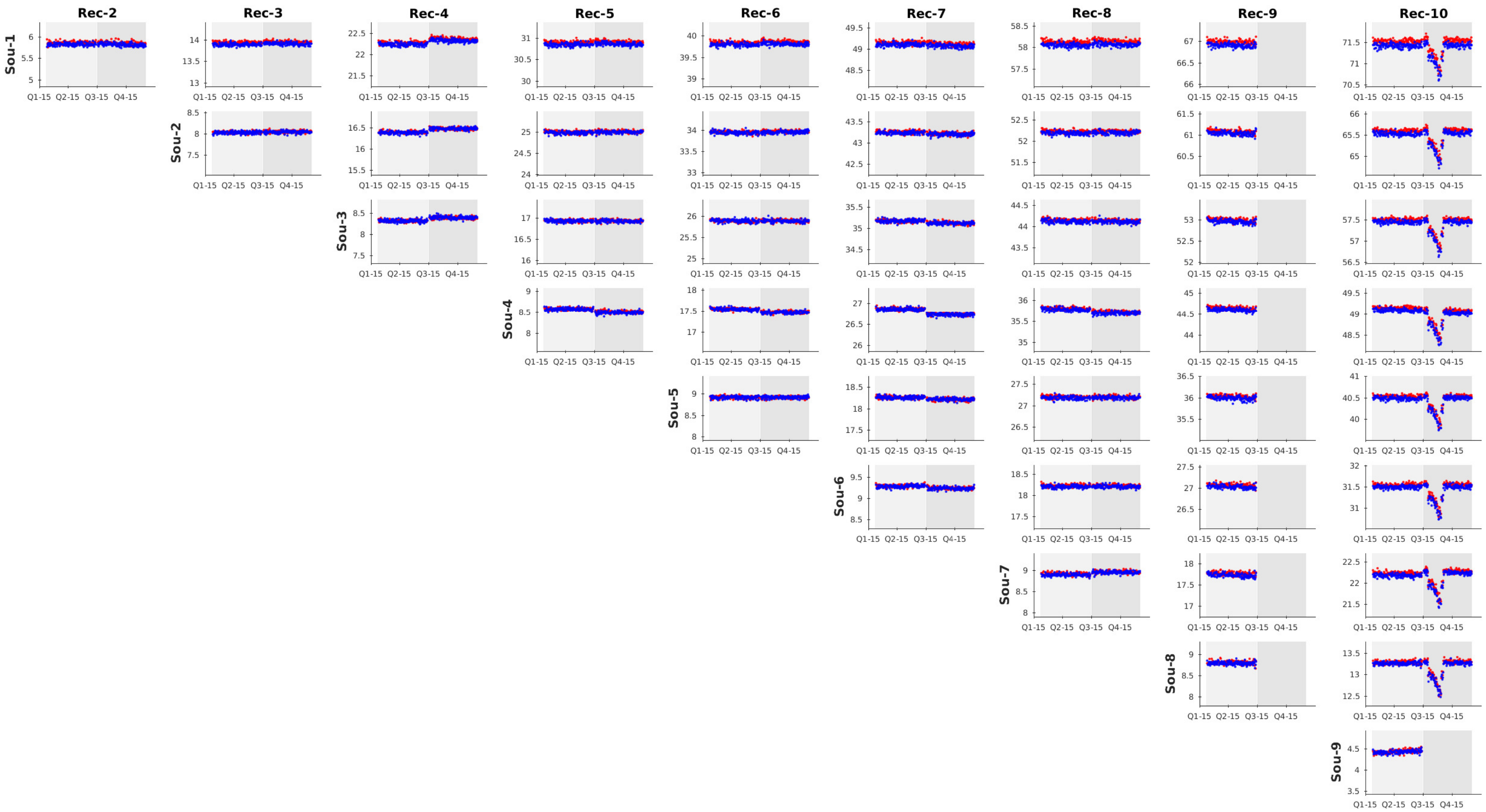

Figure S3: P-wave travel times from stacked deconvolutions per day for all geophone combinations. Rows show diagrams for geophones acting as virtual source, columns those for geophones acting as virtual receiver. The first deployment period (Jan 23 - Jun 29, 2015) has a light-grey background, the second deployment period (Jul 3 - 1 Dec 1, 2015) is in darker grey. Travel-time shifts between the two deployments are caused by small changes in geophone locations. P-wave travel times obtained for trains from Stedum to Loppersum are in blue, for Loppersum to Stedum they are in red. Travel times along the vertical axis are in ms.

2 Time-lapse borehole fluid wave travel times by deconvolution interferometry

Apart from the low-amplitude train signals (30 - 90 Hz), the geophone array also recorded intermittently occurring, high-amplitude (> 4 μm /s), high-frequency (15 - 1000 Hz) downward propagating waves (Fig. S4). Their signals are without a clear onset and the waves have an apparent velocity close to 1500 m/s, the speed of an acoustic wave in water.

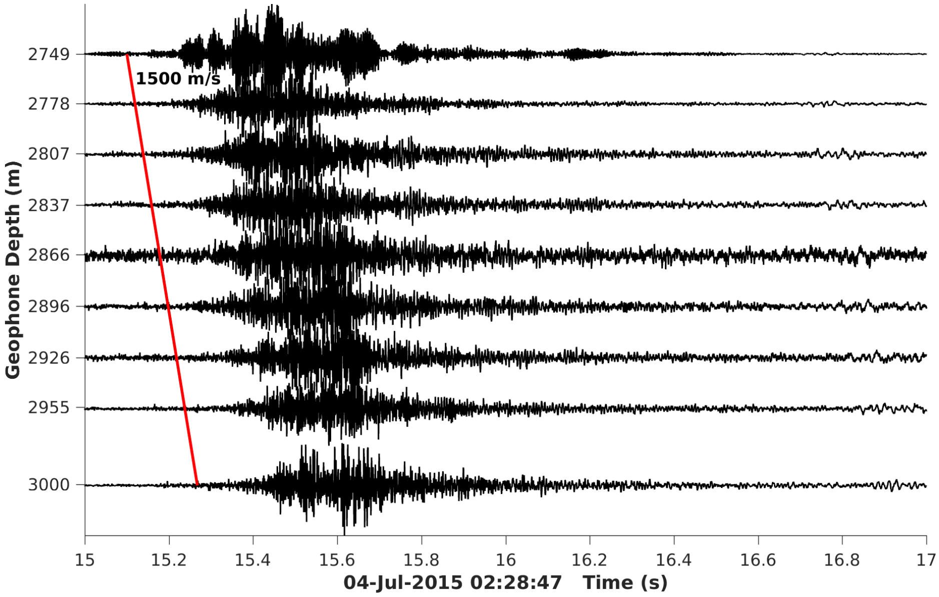

Figure S4: Example of a high-frequency borehole fluid wave.

The source of these waves is not clear and their occurrence is irregular (see below). However, their characteristics, with a propagation speed close to that of an acoustic wave in water, their high-frequency content and the absence of dispersion, suggest that they are acoustic waves that travel within the borehole fluid. Borehole fluid waves, as we call them, have previously been detected by (11) who named them tube waves. These borehole fluid waves allow independent measurements of inter-geophone travel times compared to those obtained from the train signals (sensitive to the velocity of the rock outside the well). Instrument artifacts, such as geophone movements, should affect both data sets in a similar fashion.

Vertical component spectrograms were used to detect the borehole fluid waves: the average power spectral density of all geophones in the 100-500 Hz frequency band should be higher than a certain threshold. In addition, only signals with an apparent downward velocity between 1450 and 1550 m/s were selected to exclude signals from high-frequency micro-earthquakes. We found that the number of detected

borehole fluid wave events decreased over time, starting with tens of events per day in the beginning of 2015 while later they became less frequent. During the first deployment (Jan - Jun, 2015), there were 115 days with at least one event, whereas there were only 67 days with borehole fluid wave events during the second deployment (Jul - Dec, 2015).

Similar to the train signals, we applied deconvolution interferometry to the borehole fluid waves. The deconvolution was calculated on band-pass filtered signals (100 - 300 Hz) and a 200-Hz low-pass filter was applied after that. Finally, the deconvolved responses were stacked per day to improve the signal-to-noise ratio. Fig. S5 shows the stacked responses for a day at the beginning and a day at the end of the first deployment in 2015, where the signal of the top geophone is used for deconvolution. The direct downgoing borehole fluid wave has an average speed of 1510 m/s and there are clear reflections from ~7 m below the bottom geophone and the level of the top geophone. In addition, the figure illustrates that there are time shifts between the stacked borehole fluid wave responses of February 6 (in black) and those of June 2, 2015 (in red). The time shifts are especially clear for the multiply reflected waves, with those of June 2 is being delayed. This indicates that the propagation speed of the borehole fluid wave decreased with time.

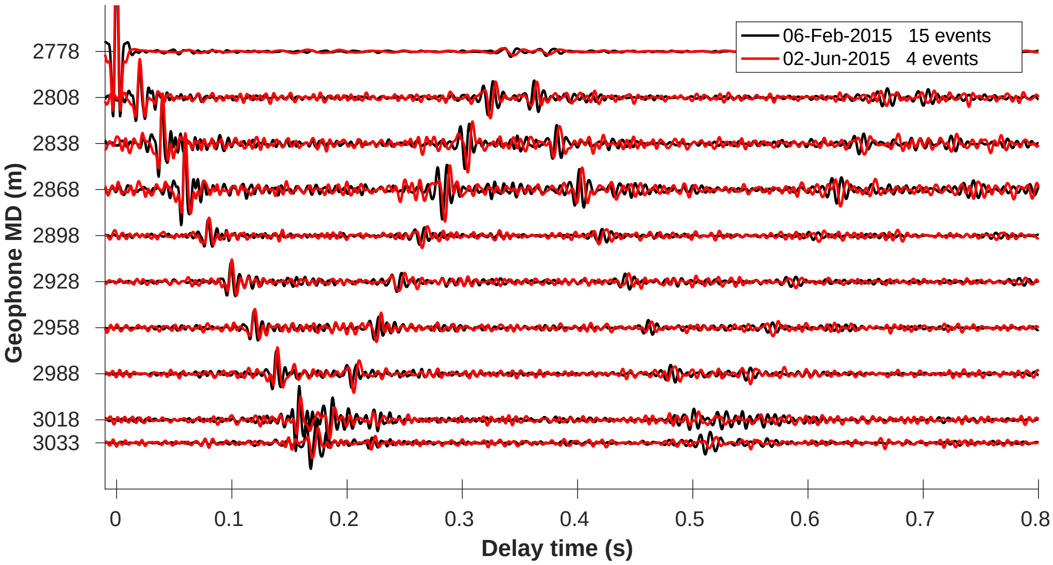

Figure S5: Stacked borehole fluid wave deconvolutions using the signal of the top geophone of February 6, 2015 (black) and June 2, 2015 (red). MD is the depth measured along the well.

To investigate the travel time variations in more detail, we measured the travel times of the direct downgoing fluid waves from the daily stacked deconvolutions. Note that the signal-to-noise ratio of these stacks depends on the (varying) amplitudes of the borehole fluid wave signals as well as on the number

of fluid wave events that were identified per day. This means that the accuracy of the measurements varies.

Fig. S6 presents the travel times of all inter-geophone borehole fluid waves. In general, the data show a trend of increasing travel times with time, and there does not seem to be a correlation with the travel times obtained by deconvolution of the train signals (Fig. S3). It is unlikely that the travel-time changes of the borehole fluid waves are related to geophone displacements, because this would require that all inter-geophone distances increase with time. Furthermore, the estimated displacements would be too large. For example, the travel time increase of the borehole fluid wave of ~1.8 ms from geophone 1 to 10 for the first 5 month deployment corresponds to a relative travel-time change (dt/t) of ~1.1%. This would translate to a ~1.1% change in distance, corresponding to a ~2.7 m elongation of the geophone string. This is not only unlikely, such an elongation would also have been detected by the travel time data from the train signal deconvolutions. Thus, it is more likely that there was a change in the properties of the borehole fluid with time. This could be caused by a temperature increase caused by heat production by the geophones as observed by (1), or by a gradual change in composition of the borehole fluid. The precise mechanism is unclear.

Finally, the interpretation of a movement of geophone 10 relative to the other geophones, to explain the travel-time data obtained from the train signals, can be discarded because the 5% (0.7 ms / 13.3 ms) decrease in travel time between geophone 8 and 10 (of 30 ms) would correspond to a systematic 1.6 ms decrease in travel time for all borehole fluid wave deconvolutions, which is not observed.

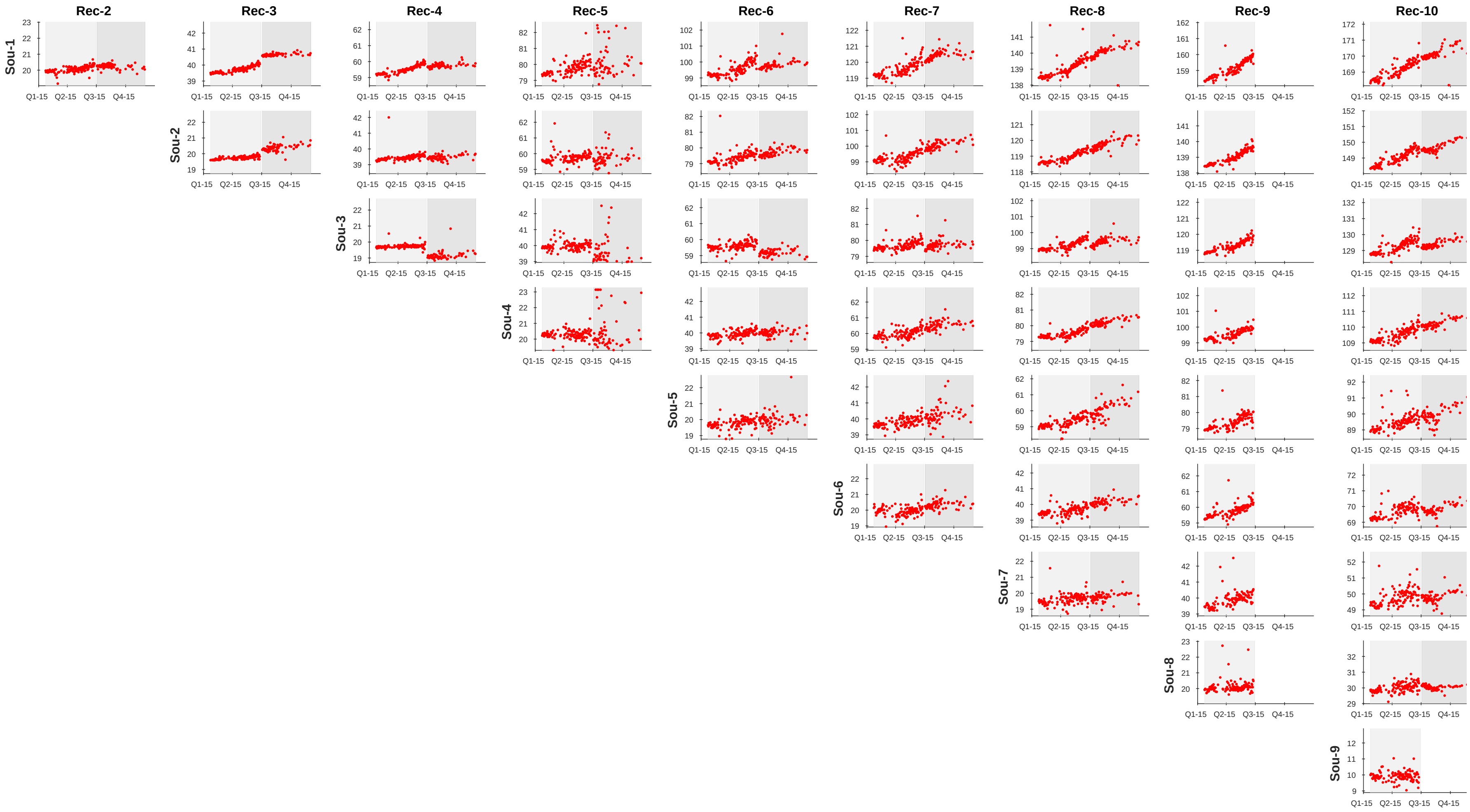

Figure S6: Similar to Fig. S3 but for inter-geophone travel times of borehole fluid waves. The travel times along the vertical axis are in ms.

3 Time-lapse P-to-S converted wave travel times by Horizontal-Vertical deconvolution of train signals

3.1 Horizontal-Vertical deconvolution: HZdecon

The receiver function method has been widely used by the seismological community to image subsurface discontinuities, mostly by employing incident P waves from teleseismic earthquakes (8). A (P-wave) receiver function is the deconvolution of the horizontal component by the vertical component for a time window that encompasses the direct P wave with a coda that includes near-receiver P-to-S converted waves (6; 5). It eliminates the source wavelet of the incoming wave and the common travel time to the interface at which conversion takes place. Contrary to the standard receiver function setting with P-wave incidence from below (from teleseismic earthquakes) and a receiver at the surface, in the current setting we have (anthropogenic) sources at the surface that are recorded by a geophone array at 3 km depth. For nearby (∼0.5 km) passing trains, the direct P waves are mainly recorded on the vertical component of the geophones, whereas the P-to-S converted waves (PS waves) from lithological interfaces above the geophone array are mainly recorded in the horizontal direction. We follow the classical receiver function approach of (5) to obtain what we call Horizontal-Vertical deconvolution, or HZdecon. For a single receiver, the time domain deconvolution of the horizontal component time segment, $H(t)$, by the vertical component, $Z(t)$, becomes a division in the frequency domain. Together with a water-level stabilization and a Gaussian low-pass filter, the HZdecon response in the frequency domain becomes

$$HZ(\omega) = \frac{H(\omega)Z^*(\omega)}{\Phi(\omega)}G(\omega) \tag{1}$$

where $Z^*(\omega)$ is the complex conjugate of $Z(\omega)$ and $\Phi(\omega)$ the vertical-component auto-correlation with a water level tuned by the parameter c.

$$\Phi(\omega) = max\{Z(\omega)Z^*(\omega),\ c \cdot max\{Z(\omega)Z^*(\omega)\}. \tag{2}$$

$G(\omega)$ is the Gaussian low-pass filter determined by frequency α,

$$G(\omega) = e^{\frac{-\omega^2}{4\alpha^2}}. \tag{3}$$

After transformation back into the time domain the HZdecons show PS (P-to-S converted) waves as arrivals at delay times relative to the direct P wave.

3.2 Synthetic HZdecons

To illustrate the method, we first applied the method to synthetic data and we then compared the results to the observed train-signal HZdecons. Here we give a summary, more details can be found in (12).

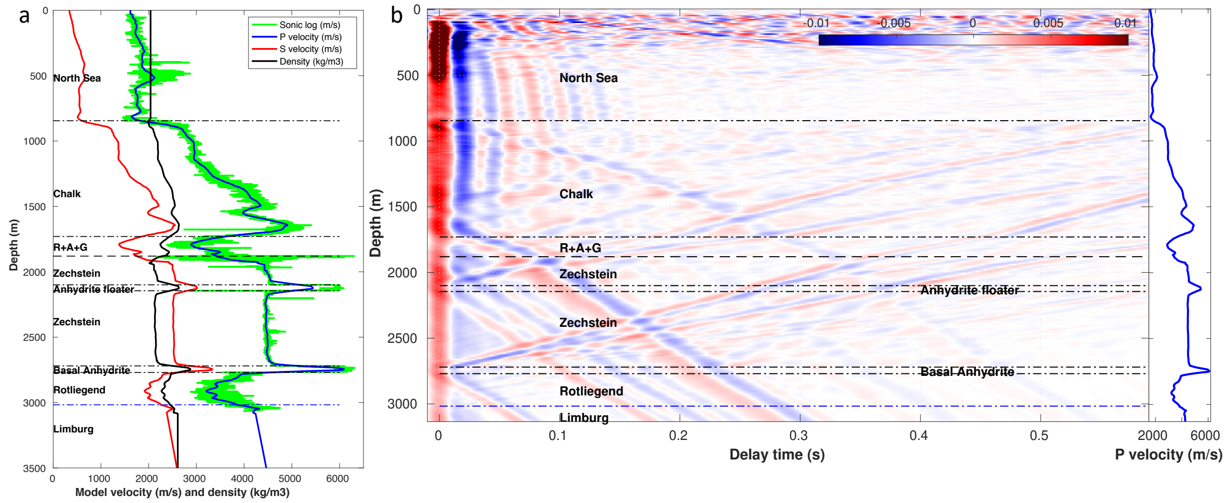

Figure S7: (a) Seismic velocity and density model derived from sonic log data of SDM-1 (green). Nomenclature of stratigraphic layers adopted from (7) and (9): North Sea group (Quaternary and Tertiary sediments), Chalk group (Late Cretaceous carbonate rocks), R+A+G (sedimentary sequence of Early Cretaceous Rijnland group, Jurassic Altena group and Triassic Germanic group), Zechstein group (rocksalt) with anhydrite layer near top and at the bottom, Rotliegend reservoir (Permian sandstone), Limburg group (Carboniferous siliciclastic deposits). (b) HZdecon results computed from SPECFEM2D synthetics for an explosive source at 20 m depth, at 500 m distance from a vertical array of receivers (0 - 3130 m depth).

The sonic log data of well SDM-1, provided by NAM, were smoothed by harmonic averaging over a 60 m sliding window to obtain a realistic, yet relatively smooth, P-wave velocity model from the surface down to the gas reservoir. The corresponding S-wave velocity and density profiles were computed from the P-wave velocities with the relations given by (7) for the various lithologies. The model is shown in Fig. S7a.

Synthetic seismograms were calculated with the spectral element code SPECFEM2D (4; 10; 3) for an explosive source at 20 m depth and at 500 m distance from a vertical array of receivers from the surface to 3130 m depth (with 10 m spacing). The HZdecon responses are displayed in Fig. S7b. They show downgoing PS waves from their conversion interfaces (starting at zero delay time with respect to the direct P wave) as well as upward reflected P-to-S converted waves.

3.3 Observed train-signal HZdecons

The stacked HZdecons of the roughly 9000 train signals (Jul. - Dec. 2015) are shown in Fig. S8 (black lines, with the synthetic HZdecons of Fig. S7b in the background). The horizontal direction was taken as the direction of the largest amplitude PS waves (131°), which roughly corresponds to the direction of minimum distance to the railway track.

The first, and also the largest PS wave, is the conversion from the basal anhydrite (0.05 s delay time at 3000 m depth). Other PS waves can be identified as well, for instance the PS wave from the top of the Zechstein rocksalt (0.2 s delay time at 3000 m depth; see Fig. S7b for the interpretation).

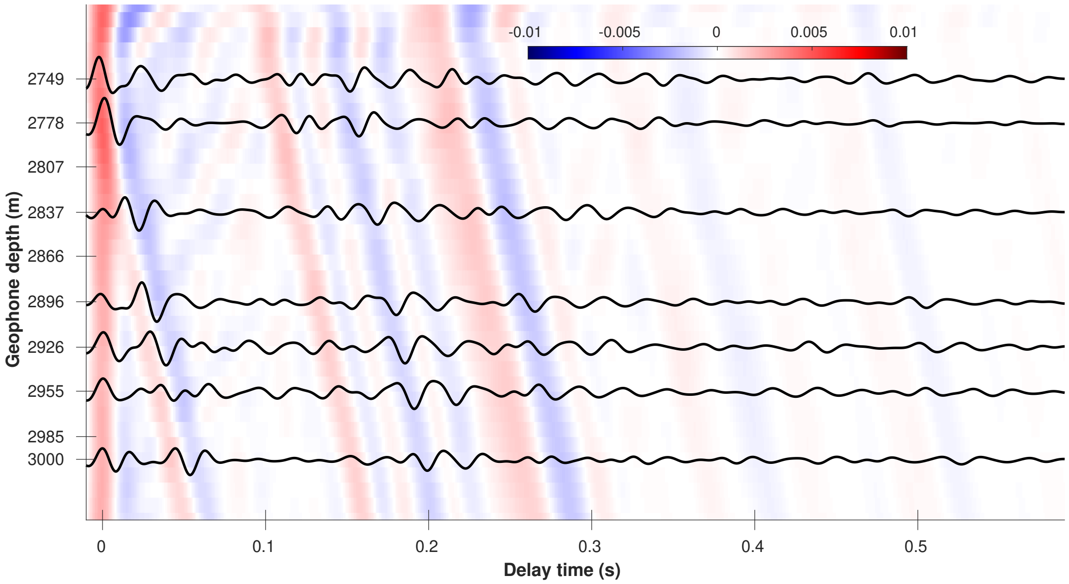

Figure S8: Stacked HZdecons of the ~9000 train signals (black lines). Synthetic HZdecons for an explosive source at 500 m distance (Fig. S7) are shown in the background.

3.4 Time-lapse train-signal HZdecons

To identify potential time-lapse changes in the HZdecons, we stacked the responses per day (~30 trains) for trains traveling in the two opposite directions. The results showed no significant travel time shifts, except for those of geophone 10 for the period from mid July to the beginning of September (see Fig.

S9a for trains from Stedum to Loppersum).

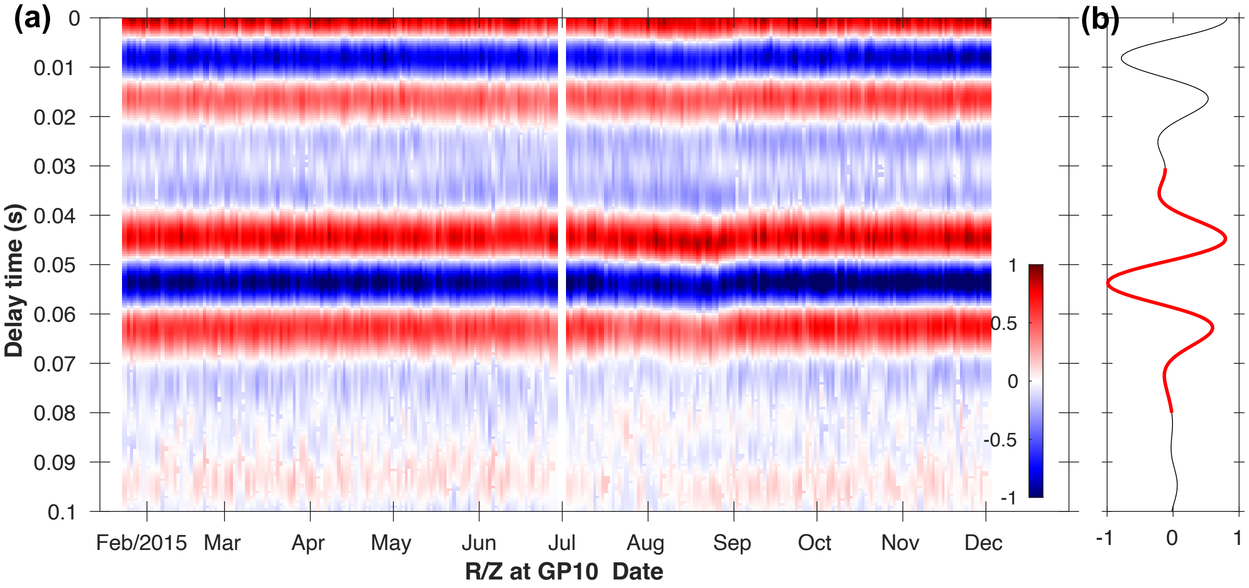

Figure S9: (a) Daily stacked HZdecons of geophone 10 for trains from Stedum to Loppersum. (b) Average HZdecon with the time window (0.03 to 0.08 s) used from cross-correlation in red.

To quantify the travel-time changes, we cross-correlated each of the (daily stacked) HZdecons with the average HZdecon. We did this for a 0.05 second time window around the PS wave from the basal anhydrite layer (red segment in Fig. S9b). The time shift is retrieved as the time of maximum cross-correlation. The cross-correlation analysis was performed for both train directions to assess the consistency of the results and to obtain an indication of the uncertainty of the measurements. The travel time data are presented in Fig. 2b of the main article.

4 Noise level and micro-earthquake

Fig. S10a shows the decrease in noise level at the beginning of the anomalous period. At the time indicated by the onset (the peak in energy density within the ellipse) a micro-earthquake occurred nearby. The 3-component seismograms of this event are shown in Fig. S10c.

This micro-earthquake may have been induced by poroelastic stressing (2), i.e. deformation of the matrix material by a pore pressure change in the fluid caused by the arrival of the pore pressure front.

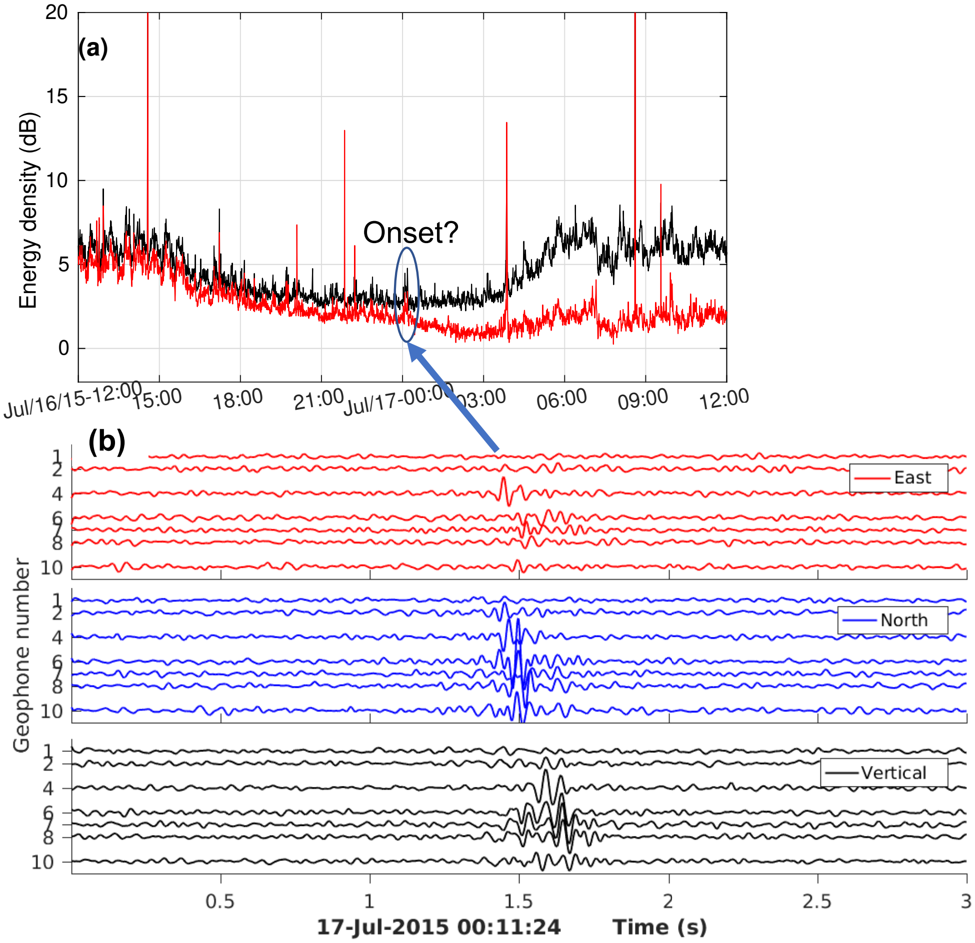

Figure S10: (a) Vertical component noise level of geophone 8 (black) and 10 (red) for the 5-30 Hz frequency band at the beginning of the anomalous period. (b) Micro-earthquake at the onset time of the anomaly. Top: East components (red). Middle: North components (blue), Bottom: Vertical components (black). The data of geophones 3, 5 and 9 are not shown because geophone 9 was out of order, as well as the horizontal components of geophones 3 and 5.

5 P-wave velocity above and below GWC

The figure below shows how the average P-wave velocity above and below the GWC was determined. The region between the dashed and solid red line (GWC) is ignored because it is the zone with reduced hydrocarbon saturation.

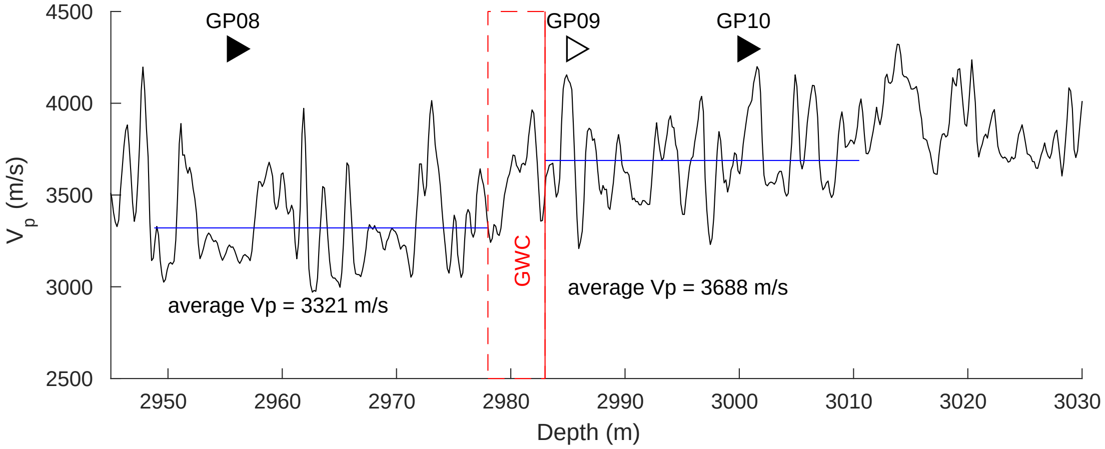

Figure S11: Determination of the average P velocity above and below the GWC from sonic log data. The region between the dashed and solid (GWC) red line is excluded because there is a gradual change in gas saturation just above the GWC.

References

[1] M. Cannon and P. Kole. The first year of distributed strain sensing (DSS) monitoring in the Groningen gas field. *NAM report*, (SR.17.009342), 2018.

[2] Kyung Won Chang and Paul Segall. Injection-induced seismicity on basement faults including poroelastic stressing. *Journal of Geophysical Research: Solid Earth*, 121(4):2708–2726, 2016.

[3] Dimitri Komatitsch, Gordon Erlebacher, Dominik Göddeke, and David Michéa. High-order finite-element seismic wave propagation modeling with MPI on a large GPU cluster. *Journal of Computational Physics*, 229(20):7692–7714, 2010.

[4] Dimitri Komatitsch, Jeroen Ritsema, and Jeroen Tromp. The spectral-element method, Beowulf computing, and global seismology. *Science*, 298(5599):1737–1742, 2002.

[5] C.A. Langston. Structure under Mount Rainier, Washington, inferred from teleseismic body waves. *Journal of Geophysical Research*, 84(B9):4749–4762, 1979.

[6] Robert A Phinney. Structure of the Earth's crust from spectral behavior of long-period body waves. *Journal of Geophysical Research*, 69(14):2997–3017, 1964.

[7] R. Romijn. Groningen velocity model 2017 - Groningen full elastic velocity model. *NAM report*, 2017.

[8] Stéphane Rondenay. Upper mantle imaging with array recordings of converted and scattered teleseismic waves. *Surveys in geophysics*, 30(4):377–405, 2009.

[9] TNO - Geologische Dienst Nederland. Stratigrafische nomenclator van Nederland. http://www.dinoloket.nl/stratigrafische-nomenclator, 2022.

[10] Jeroen Tromp, Dimitri Komatitsch, and Qinya Liu. Spectral-element and adjoint methods in seismology. *Communications in Computational Physics*, 3(1):1–32, 2008.

[11] Yoones Vaezi and Mirko van der Baan. Interferometric assessment of clamping quality of borehole geophones. *Geophysics*, 80(6):WC89–WC98, 2015.

[12] Wen Zhou. *Investigating the Groningen gas reservoir: From passive seismic monitoring to experiments on effects of pore pressure on fault slip.* PhD thesis, Utrecht University, 2020.

[13] Wen Zhou and Hanneke Paulssen. Compaction of the Groningen gas reservoir investigated with train noise. *Geophysical Journal International*, 223(2):1327–1337, 2020.
